# Risk Factors for Late-Life Cognitive Decline and Variation with Age and Sex in the Sydney Memory and Ageing Study

**DOI:** 10.1371/journal.pone.0065841

**Published:** 2013-06-14

**Authors:** Darren M. Lipnicki, Perminder S. Sachdev, John Crawford, Simone Reppermund, Nicole A. Kochan, Julian N. Trollor, Brian Draper, Melissa J. Slavin, Kristan Kang, Ora Lux, Karen A. Mather, Henry Brodaty

**Affiliations:** 1 Centre for Healthy Brain Ageing, School of Psychiatry, Faculty of Medicine, University of New South Wales, Australia; 2 Neuropsychiatric Institute, Prince of Wales Hospital, New South Wales, Australia; 3 Primary Dementia Collaborative Research Centre, School of Psychiatry, Faculty of Medicine, University of New South Wales, Australia; 4 Department of Developmental Disability Neuropsychiatry, School of Psychiatry, Faculty of Medicine, University of New South Wales, Australia; 5 Academic Department for Old Age Psychiatry, Prince of Wales Hospital, New South Wales, Australia; 6 South-Eastern Area Laboratory Services, Prince of Wales Hospital, New South Wales, Australia; Nathan Kline Institute and New York University School of Medicine, United States of America

## Abstract

**Introduction:**

An aging population brings increasing burdens and costs to individuals and society arising from late-life cognitive decline, the causes of which are unclear. We aimed to identify factors predicting late-life cognitive decline.

**Methods:**

Participants were 889 community-dwelling 70–90-year-olds from the Sydney Memory and Ageing Study with comprehensive neuropsychological assessments at baseline and a 2-year follow-up and initially without dementia. Cognitive decline was considered as incident mild cognitive impairment (MCI) or dementia, as well as decreases in attention/processing speed, executive function, memory, and global cognition. Associations with baseline demographic, lifestyle, health and medical factors were determined.

**Results:**

All cognitive measures showed decline and 14% of participants developed incident MCI or dementia. Across all participants, risk factors for decline included older age and poorer smelling ability most prominently, but also more education, history of depression, being male, higher homocysteine, coronary artery disease, arthritis, low health status, and stroke. Protective factors included marriage, kidney disease, and antidepressant use. For some of these factors the association varied with age or differed between men and women. Additional risk and protective factors that were strictly age- and/or sex-dependent were also identified. We found salient population attributable risks (8.7–49.5%) for older age, being male or unmarried, poor smelling ability, coronary artery disease, arthritis, stroke, and high homocysteine.

**Discussion:**

Preventing or treating conditions typically associated with aging might reduce population-wide late-life cognitive decline. Interventions tailored to particular age and sex groups may offer further benefits.

## Introduction

Late-life cognitive decline ranges from normal age-related change at its mildest, through mild cognitive impairment (MCI), to dementia at its most severe. While the personal and societal impacts of dementia are substantial, even relatively mild cognitive decline can generate significant levels of functional dependence [1] and reduce quality of life. Mitigating cognitive decline requires knowing its causes, but a recent systematic review [2] found the evidence for putative risk and protective factors reported by observational studies to be inconsistent or only poorly supported. This could be because associations with cognitive decline are complicated by effects that vary with age [2], sex [3], [4] and cognitive measure [5]–[8].

We recently reported that age and sex influenced cross-sectional risk profiles for MCI and its subtypes among population-based elderly individuals [9], [10]. The current study used the same sample to identify factors longitudinally associated with late-life cognitive decline. A focus on effects varying with age or sex was maintained, and we again investigated a broad range of factors that included sociodemographic characteristics and lifestyle, medications, cardiac, physical, mental and general health, and biochemical indices of health. Importantly, this included a number of factors frequently associated with poorer cognition in the elderly, including *apolipoprotein E* (*APOE*) ε4 status [2] and smelling ability [8], [11]–[13]. Cognitive decline was defined as worsening performance on measures addressing attention/processing speed, executive function, memory and global ability, and as a diagnosis of incident MCI or dementia. For significant associations between factors and decline we calculated the population attributable risk (PAR). This statistic considers prevalence in addition to strength of association, and may help identify interventions offering the greatest population-wide benefits.

## Methods

### Participants

Participants were from the longitudinal, community-based Sydney Memory and Ageing Study (MAS), described in detail elsewhere [14]. Briefly, the overall sample comprised 1037 individuals recruited randomly through the electoral roll who were 70–90 years old, non-demented, living in the community, and sufficiently competent in English to complete assessments. Exclusion criteria included a history of psychosis, intellectual handicap, multiple sclerosis, motor neuron disease, progressive malignancy, a Mini-Mental State Examination [15] score <24 after adjustment for age, non-English speaking background and education [16], and a diagnosis of dementia. All participants were assessed at baseline, and 889 assessed again at a 2-year follow-up (mean duration 23 months, 12 days). Of those not assessed at follow-up, 43 were deceased and 105 declined to participate.

### Ethics Statement

The study was approved by the ethics committees of the University of New South Wales and the South Eastern Sydney and Illawarra Area Health Service. Written, informed consent was obtained from each participant.

### Cognitive Domain and Global Cognition Scores

Trained psychology graduates administered a battery of neuropsychological tests from particular cognitive domains: attention/processing speed (Digit-Symbol Coding [17], Trail Making Test A [18]), memory (Logical Memory Story A [19], Rey Auditory Verbal Learning Test [18], Benton Visual Retention Test [20]), language (Boston Naming Test [21], Semantic Fluency [18]), and executive function (Controlled Oral Word Association Test [18], Trail Making Test B [18]). A more detailed account is given elsewhere [14].

Raw test scores were transformed to *z*-scores using the baseline means and SDs of a subgroup having fluent spoken English before the age of 10 years and classified as cognitively normal at baseline (n = 504). Domain scores were calculated by averaging the *z*-scores of component tests, and subsequently transformed so the means and SDs of the cognitively normal subgroup were 0 and 1. Global cognition scores were calculated by averaging across the domain scores (provided that no more than one of these was missing), and then similarly transformed.

### Cognitive Status

Consensus diagnoses of MCI were made by a panel of psychogeriatricians, neuropsychiatrists and clinical and research neuropsychologists using current international consensus criteria [22]. MCI was diagnosed in individuals meeting all of: self or informant complaint of memory or other cognitive function decline; objective cognitive impairment (at least one test score 1.5 SD or more below published normative values, adjusted for age and/or education where possible); no dementia on the basis of DSM-IV criteria [23]; and no or minimal impairment in instrumental activities of daily living attributable to cognitive impairment (total average score <3.0 on the Bayer Activity of Daily Living (ADL) Scale [24] adjusted for physical impairment). Participants not classified as having MCI or dementia were deemed to have normal cognition.

### Risk and Protective Factors

Table 1 shows the putative risk and protective factors investigated. Some data were obtained via interviews and questionnaires, including the 15-item Geriatric Depression Scale [25] and in most cases (93.9%) supplemented by an informant. Physical examinations by trained research assistants included measures of seated blood pressure, height and weight, a 6-m timed walk [26], and the Brief Smell Identification Test [27]. Venous blood was obtained after overnight fasting and stored in aliquots frozen at −80°C, with total cholesterol, homocysteine and creatinine measured from heparin plasma aliquots using a Beckman LX20 Analyser by a timed-endpoint method (Fullerton, California, USA), EDTA plasma aliquots using reverse phase HPLC with fluorometric detection after derivatization with 4-aminosulfonyl-7-fluorobenzo-2-oxa1,3-diazole (coefficient of variation = 6.7% at 11.7 µmol/L and 6.0% at 30.0 µmol/L) (BioRad Munich, Germany) and the Jaffe rate method, respectively. Standard procedures were used for *APOE* genotyping, as previously described [14].

**Table 1:** Baseline characteristics of the study participants^a^.

Characteristic	Whole sample^b^	Women	Men	Age association^c^
***Sociodemographic***				
Age, mean (SD), y	78.59 (4.75)	78.60 (4.87)	78.59 (4.60)	–
Males	408 (45.9)	–	–	d = 0.01
Education, mean (SD), y	11.68 (3.49)	11.13 (3.05)	12.33 (3.85)*	r = –0.035
Married	43.0	134 (27.9)	247 (60.7)*	d = –1.28*
NESB	16.2	71 (14.8)	73 (17.9)	d = 1.17*
***Cardiac health***				
Hypertension	738 (83.0)	382 (79.4)	356 (87.3)*	d = 1.14*
Antihypertensives	522 (41.3)	266 (55.3)	256 (62.7)*	d = 0.73*
Coronary artery disease	166 (18.7)	56 (11.6)	110 (27.0)*	d = 1.86
Atrial fibrillation	53 (6.0)	19 (4.0)	34 (8.5)*	d = –0.67
Other heart disease	102 (11.5)	42 (8.7)	60 (14.7)*	d = 0.84
Systolic BP, mean (SD), mmHg	144.82 (20.22)	143.07 (20.45)	146.85 (19.77)*	r = 0.141*
Diastolic BP, mean (SD), mmHg	82.07 (10.71)	81.88 (10.06)	82.28 (11.42)	r = –0.055
***Physical health***				
BMI, mean (SD), kg/m^2^	27.10 (4.46)	26.68 (4.65)	27.59 (4.17)*	r = –0.123*
Diabetes	128 (14.40	47 (9.8)	81 (19.9)*	d = 0.26
Hypoglycemics	78 (8.8)	27 (5.6)	51 (12.5)*	d = –0.45
Hypercholesterolemia	536 (60.5)	290 (60.4)	246 (60.6)	d = –0.88*
Hypolipidemics	457 (51.4)	226 (47.0)	231 (56.6)*	d = –0.30
Stroke	35 (4.0)	12 (2.5)	23 (5.7)*	d = 0.13
Migraines	137 (15.4)	104 (21.7)	33 (8.1)*	d = –1.23*
Kidney disease	20 (2.3)	4 (0.8)	16 (3.9)*	d = 1.67
Arthritis	480 (54.5)	275 (57.8)	205 (50.7)*	d = –0.41
Apnea	47 (5.3)	14 (2.9)	33 (8.1)	d = –0.55
Anemia	110 (12.4)	84 (17.6)	26 (6.4)*	d = 0.33
***Mental health***				
GDS score, mean (SD)	2.18 (1.97)	2.05 (1.75)	2.33 (2.18)*	r = 0.133*
History of depression	133 (15.0)	80 (16.6)	53 (13.0)	d = –0.90*
Antidepressants	82 (9.2)	54 (11.2)	28 (6.9)*	d = –0.03
Antianxiety agents	41 (4.6)	29 (6.0)	12 (2.9)*	d = 1.80*
**Lifestyle**				
Alcohol drinker	783 (88.2)	407 (84.8)	376 (92.2)*	d = –0.70
Ever smoker	478 (53.9)	203 (42.3)	275 (67.6)*	d = 0.24
Mental activity, mean (SD)	2.53 (0.86)	2.52 (0.86)	2.53 (0.86)	r = –0.152*
Physical activity, mean (SD)	1.66 (1.13)	1.57 (1.12)	1.77 (1.14)*	r = –0.208*
Low social activity	116 (13.4)	56 (11.9)	60 (15.2)	d = 0.26
***General health***				
Low health status	132 (14.9)	64 (13.3)	68 (16.7)	d = 0.74
6-m walk time, mean (SD), s	9.12 (2.81)	9.36 (3.07)	8.84 (2.44)*	r = 0.296*
BSIT score, mean (SD)	9.30 (2.13)	9.72 (1.79)	8.81 (2.39)*	r = –0.207*
Inadequate vision	82 (9.3)	51 (10.7)	31 (7.6)	d = 0.67
***Laboratory measures***				
*APOE* ε4 carrier	197 (22.6)	105 (22.3)	92 (22.8)	d = –0.77*
Homocysteine, mean (SD), µmol/L	11.34 (3.96)	10.62 (4.01)	12.13 (3.76)*	r = 0.243*
Cholesterol, mean (SD), mmol/L	4.73 (0.99)	4.97 (0.98)	4.45 (0.93)*	r = –0.020
Low eGFR	296 (35.6)	166 (37.6)	130 (33.3)	d = 2.33*

*APOE* = *apolipoprotein E*; BMI = body mass index; BP = blood pressure; BSIT = Brief Smell Identification Test; eGFR = estimated glomerular filtration rate; GDS = Geriatric Depression Scale; NESB = non-English-speaking background.

a Data are presented as No. (%), unless otherwise indicated.

b Maximum n for the whole sample is 889; minimum is 820 (homocysteine).

c For categorical factors, d is the difference in mean age of those meeting the factor versus those not meeting the factor. For continuous factors, r is the correlation coefficient with age.

* *p*<.05 for men versus women (t- or χ^2^ tests) or age associations (t- tests or Pearson correlations).

Our factor definitions included the following. Non-English-speaking background: not speaking fluent English before 10 years old. Hypertension: previous diagnosis and current treatment, or either systolic blood pressure ≥140 mmHg or diastolic blood pressure ≥90 mmHg (as per JNC-7 values) [28]. Coronary artery disease: previous diagnosis of heart attack or angina. Other heart disease: previous diagnosis of cardiac arrhythmia, cardiomyopathy, or heart valve disease. Diabetes: declaration of, or taking medication for, diabetes, or fasting blood glucose >7.0 mmol/L. History of depression: previous diagnosis and treatment. Mental activity: average days/week of current participation in 13 listed activities (e.g., reading books). Physical activity: sum of current participation across 8 listed activities (e.g., bicycling), any valid other activity (e.g., yoga), and walking. Social activity: average number of current face-to-face contacts with friends or relatives per month; categorised as low if <5. Health status: self-reported as poor, fair, good, very good or excellent; categorised as low if either poor or fair. Alcohol drinker: drinking at least “monthly or less” rather than “not at all” in the past year. Ever smoker: past or current smoker. Vision: self-reported as adequate or inadequate for all purposes with glasses on. Estimated glomerular filtration rate (eGFR): calculated as 175 × (serum creatinine [µmol/L] × 0.0113)^−1.154^ × age^−0.203^, further multiplied by 0.742 for females; categorised as low if <60 ml/min/1.73m^2^.

### Statistical Analysis

Descriptive statistics were computed, sex comparisons made using either t*-* or χ*^2^* tests, and associations with age investigated using Pearson correlations or t-tests. For each cognitive performance measure, general linear models (GLMs) adjusted for age and sex were used to identify baseline factors associated with longitudinal change, calculated as follow-up *z*-scores minus baseline *z*-scores. Factors with *p*<0.1 were entered into a multivariable GLM that included age and sex if not already present, and that was reduced stepwise until only factors with *p*<0.05 remained (significant main effect factors). For all baseline factors, we also ran a GLM containing age and sex interaction terms. Each interaction term with *p*<0.1 was included in a multivariable GLM containing the significant main effect factors plus age (for sex interactions) or sex (for age interactions). Subsequently significant sex interactions (*p*<0.05) were further explored with stratified GLM analyses (adjusted for age) to determine the nature of association within each of men and women. Associations between baseline factors and incident MCI or dementia were investigated with a similar series of analyses, using logistic regression rather than GLMs.

PARs were calculated for all factors independently associated with cognitive decline or incident MCI or dementia. Decline was categorized as less severe or more severe, representing a decrease in *z*-score from baseline to follow-up of >1 and >1.5, respectively. Values for factors measured as continuous variables were categorized into two groups. We used the PAR formula: PAR = 1– (1/*x*)∑*_i_*(1/r*_i_*), where *x* is the number of cases and r*_i_* is the multivariable-adjusted odds ratio (OR) corresponding to the exposure of case *i* to one or more risk factors [29]. The ORs for calculating PARs were obtained from logistic regressions containing all factors independently associated with the outcome (and age or sex if not already present or redundant). PARs were considered salient if the 95% confidence interval (CI) of the corresponding OR did not include 1. All analyses were performed using IBM SPSS Statistics 20.

## Results

### Sample Characteristics

Participant characteristics, sex comparisons and age associations are shown in Table 1. The mean (SD) age of the sample was 78.59 (4.8) years and 45.9% were male. Men and women differed significantly on most factors, including heart disease and diabetes being more prevalent in men and cholesterol levels higher in women. Age was associated with many factors, including a positive correlation with 6-m walk time.

### Cognitive Decline from Baseline to Follow-up

Table 2 shows mean cognitive performance changes between baseline and follow-up. Performance declined significantly for all cognitive measures across the whole sample and within each sex subgroup. Older age was associated with greater cognitive decline, but men and women did not differ. The cognitive status of 665 participants could be classified at both baseline and follow-up, with 77 new cases of MCI and 16 new cases of dementia at follow-up. Cases of incident MCI or dementia tended to be older (baseline age 79.1±4.8 years versus 78.2±4.6 years; t = 1.79, *p* = 0.074) and were more frequent among men than women (54/296, 18.2% vs. 39/369, 10.6%; χ*^2^* = 8.04, *p* = 0.005).

**Table 2:** Change in cognitive performance^a^.

Measure	Whole sample		Women		Men		Correlation with age^b^	
	Mean (SD)	n	Mean (SD)	n	Mean (SD)	n	r	*p*
Attention/PS	−0.20 (1.01)	838	−0.17 (0.92)	449	−0.24 (1.11)	389	−0.100	.004
Executive	−0.27 (1.07)	769	−0.23 (0.96)	413	−0.31 (1.19)	388	−0.142	<.001
Memory	−0.16 (0.78)	847	−0.14 (0.75)	459	−0.19 (0.81)	388	−0.155	<.001
Global	−0.27 (0.77)	865	−0.24 (0.66)	466	−0.30 (0.88)	399	−0.149	<.001

PS = processing speed.

a Values are presented as follow-up *z*-scores minus baseline *z*-scores; all are significantly less than zero (*p*<.001).

b Pearson correlations using the whole sample.

### Factors Associated with Cognitive Decline

#### Risk factors

Table 3 shows the factors significantly associated with cognitive decline and incident MCI or dementia. Factors differed among the outcome measures, but poorer smelling ability and older age featured frequently as risk factors. Poorer smelling ability was associated with increased chances of incident MCI or dementia and greater declines in executive function, memory and global cognition. Older age predicted greater declines in memory, attention/processing speed and global cognition. Older age also emerged as a risk factor in specific subgroups (interaction results in Table 3); it was associated with greater declines in global cognition among participants with history of stroke, in attention/processing speed among migraine sufferers, and in memory among participants with coronary artery disease. Being male was associated with an increased risk of incident MCI or dementia. There were also risk factors identified as specific to men. These included an increased risk of incident MCI or dementia in men with more physical activity (B = 0.366, SE = 0.132, *p* = 0.006) and significant decline in memory if having ever smoked (B = −0.190, SE = 0.088, *p* = 0.031). Conversely, poorer smelling ability was a risk factor for incident MCI or dementia primarily in women (B = −0.281, SE = 0.082, *p* = 0.001).

**Table 3:** Baseline factors associated with cognitive decline^a^.

Outcome and factor	B	SE	*p*
***Attention/Processing speed***			
Age	−0.024	0.007	.002
Education	−0.023	0.010	.02
History of depression	−0.236	0.098	.02
Migraines × Age	−0.051	0.021	.02
Antidepressants × Sex	0.653	0.253	.01
Inadequate vision × Sex	0.624	0.269	.02
***Executive function***			
Married	0.193	0.076	.01
BSIT score	0.113	0.018	<.001
Homocysteine	−0.024	0.010	.01
Low health status × Sex	0.573	0.225	.01
***Memory***			
Age	−0.024	0.006	<.001
Coronary artery disease	−0.138	0.070	.05
Kidney disease	0.540	0.177	.002
Arthritis	−0.137	0.053	.01
Antidepressants	0.254	0.093	.007
Low health status	−0.157	0.077	.04
BSIT score	0.027	0.013	.04
History of depression × Age	0.034	0.017	.04
Coronary artery disease × Age	−0.035	0.015	.02
Diastolic blood pressure × Sex	−0.011	0.005	.03
Ever smoker × Sex	−0.246	0.111	.03
***Global cognition***			
Age	−0.019	0.006	.001
BSIT score	0.057	0.013	<.001
Stroke	−0.273	0.131	.04
Stroke × Age	−0.063	0.26	.02
Antidepressants × Age	0.039	0.019	.04
Low social activity × Age	−0.042	0.016	.008
Low eGFR × Sex	0.218	0.109	.05
***Incident MCI or dementia***			
Sex	0.770	0.252	.002
Married	−0.571	0.256	.03
BSIT score	−0.140	0.050	.005
BSIT score × Sex	0.241	0.103	.02
Diabetes × Sex	−1.821	0.743	.01
Physical activity × Sex	0.526	0.216	.02
Low eGFR × Sex	−1.362	0.524	.009
Low eGFR × Age	−0.114	0.054	.04

BSIT = Brief Smell Identification Test; eGFR = estimated glomerular filtration rate; MCI = mild cognitive impairment.

a Includes factors remaining in reduced generalized linear models initially containing all associated with the cognitive measure at *p*<.1 (including age and sex if not already present). Interaction results are adjusted for the independent factors listed, in addition to age (sex interactions) or sex (age interactions). Sex is coded as female = 0 and male = 1.

#### Protective factors

Marriage protected against declines in executive function and reduced the risk of incident MCI or dementia, whereas both kidney disease and antidepressant use were associated with less memory decline (Table 3). Sex-specific protective factors were found, with men being at reduced risk of incident MCI or dementia if having either diabetes (B = −1.184, SE = 0.550, *p* = 0.031) or low eGFR (B = −0.803, SE = 0.385, *p* = 0.037). Men also showed less decline in attention/processing speed if taking antidepressants (B = 0.628, SE = 0.241, *p* = 0.010) and in executive function if reporting low health status (B = 0.431, SE = 0.167, *p* = 0.011). Some protective factors were age-related (interaction results in Table 3). Older age was associated with less global cognitive decline among participants taking antidepressants or those reporting more social activity. Older age was also associated with less memory decline among participants with history of depression, and with reduced chances of incident MCI or dementia among participants with low eGFR.

#### Additional factors that varied with sex

Table 3 shows associations with cognitive decline that differed between men and women but were not significant within either of these: diastolic blood pressure and memory, low eGFR and global cognition, and visual adequacy and attention/processing speed.

### Population Attributable Risks

**Figure 1:**
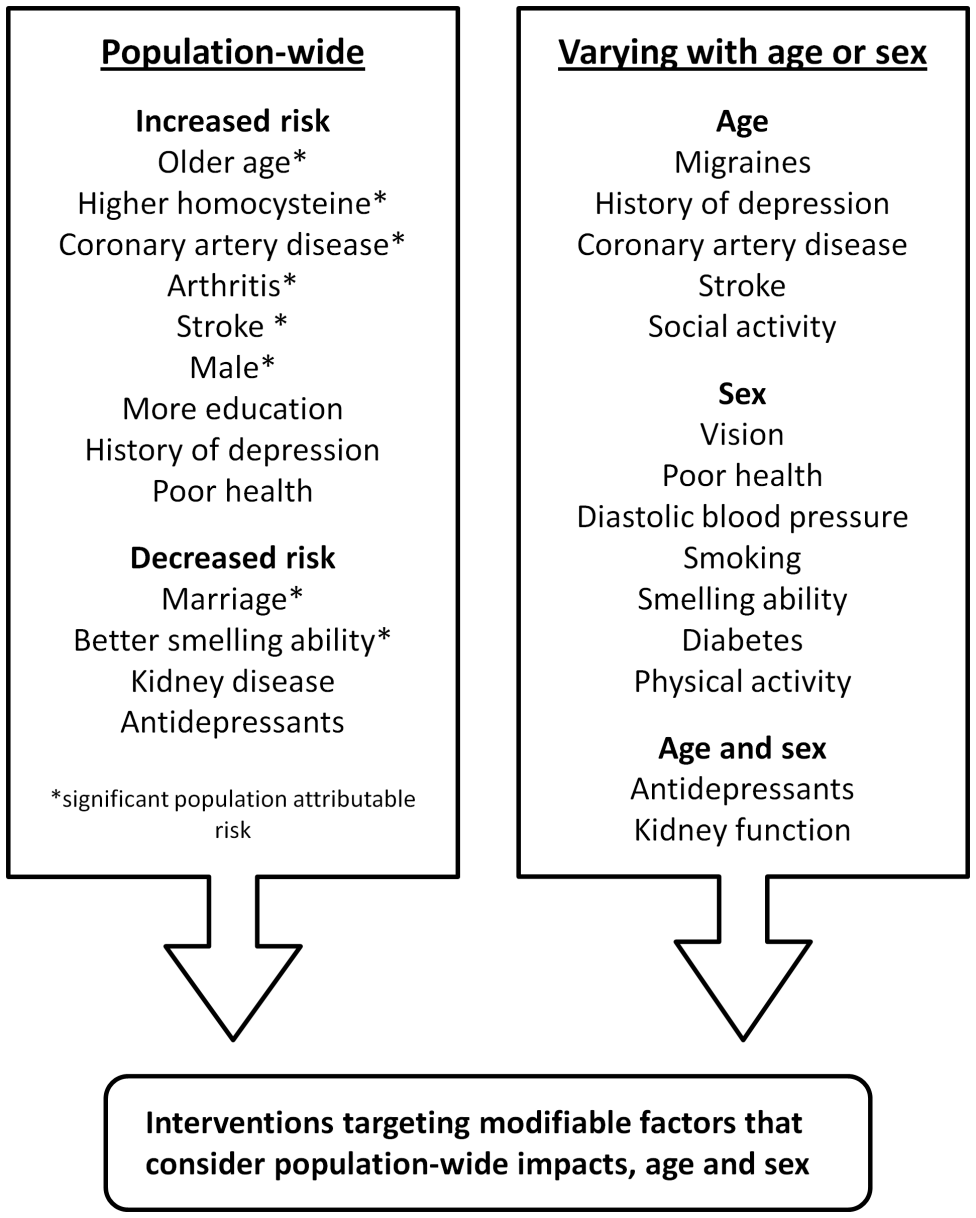
Factors associated with cognitive decline. Interventions modifying factors with a significant population attributable risk might greatly reduce population-wide cognitive decline. Age and sex interactions suggest further benefits by tailoring interventions to particular demographic groups.

The PARs (and corresponding ORs and 95% CIs) for baseline factors independently associated with incident MCI or dementia are shown in Table 4. Risks attributable to either being male (PAR = 31.3%) or having poorer smelling ability (PAR = 18.3%) were offset to at least some degree by marriage (PAR = –24.6%). The PARs of factors associated with a decline in cognitive performance are detailed in Table S1, with only the salient results outlined here. Older age (>81 years) had impacts on both less and more severe decline in global cognition (PAR = 24.4% and 27.7%), attention/processing speed (PAR = 24.2% and 21.4%), and memory (PAR = 30.5% and 49.5%). Other salient impacts included coronary artery disease on more severe memory decline (PAR = 30.1%), arthritis and poorer smelling ability on less severe memory decline (PAR = 29.1% and 17.3%, respectively), stroke on more severe global cognitive decline (PAR = 8.7%), and high homocysteine level on less severe executive function decline (PAR = 9.7%). The results of our study are summarized in Figure 1, which shows all factors significantly associated with at least one outcome measure and indicates those with a salient PAR.

**Table 4:** PARs for incident MCI or dementia^a^.

Factor	Cases; Controls (No.)	OR (95% CI)	PAR (%)
***Sex***			
Female	39; 330	1.00 (ref)	
Male	54; 242	2.17 (1.32–3.56)	31.3
***Married***			
No	32; 245	1.00 (ref)	
Yes	60; 326	0.59 (0.35–0.97)	−24.6
***BSIT score***			
≥9	56; 440	1.00 (ref)	
<9	37; 132	1.86 (1.15–3.01)	18.3

BSIT = Brief Smell Identification Test; CI = confidence interval; MCI = mild cognitive impairment; OR = odds ratio; PAR = population attributable risk.

a Model contains sex, marital status, BSIT score, and age.

### Missing Participants

We conducted subsidiary analyses comparing participants lost to follow-up with those remaining in the study to see if selective attrition could help explain unexpected findings. Compared to men remaining in the study, men lost to follow-up reported less baseline physical activity (1.37±0.89 vs. 1.77±1.14, t = 2.42, *p = *0.016) and higher rates of low health status (28.1% vs. 16.7%, χ*^2^* = 4.31, *p* = 0.038). Low eGFR tended to be more common among individuals lost to follow-up than among those remaining in the study (45.1% vs. 35.6%, χ*^2^* = 3.52, *p = *0.065).

## Discussion

We previously reported cross-sectional studies of the sample used in the current study that found risk profiles for MCI and its subtypes varied with age and sex [9], [10]. In being longitudinal, the current study allows for a more refined interpretation of factors as either increasing or decreasing the risk of cognitive decline. We have also extended upon our earlier work by investigating decline within particular cognitive domains.

### Risk and Protective Factors for Late-life Cognitive Decline

A number of risk and protective factors for late-life cognitive decline were identified, with the most prominent being age and smelling ability (others are summarized in Figure 1). Older age predicted greater declines in attention/processing speed, memory and global cognition, and poorer smelling ability predicted greater declines in executive function, memory, and global cognition, as well as the development of incident MCI or dementia. This is consistent with previous reports of both age [30] and poorer smelling ability [8], [11]–[13] being associated with cognitive decline. Many of our findings are also consistent with expectation and/or known mechanisms. For example, the association between poorer smelling ability and cognitive decline is readily accounted for by links between degenerative olfactory changes and cortical neurodegeneration [31].

### Variation with Sex and Age

The current study found a number of associations with cognitive decline that varied with age or sex (see Figure 1). Our results suggest that normal aging processes can compound the effects of factors reported elsewhere as promoting cognitive decline, including stroke [32] and low social activity [7]. Most of the variations with sex entailed a significant association in men but not women. Sex differences could reflect discrepancies in the presence or extent of various risk and protective factors and/or prevalence of outcomes. Men in our study had higher rates of incident MCI or dementia, and differed from women on most of the factors investigated, including more frequent heart disease and diabetes, but lower levels of cholesterol.

### Null and Unexpected Findings

Some measures not associated with cognitive decline in the present study are reported to be associated with decline elsewhere. This includes the *APOE* ε4 allele, which is a known risk factor for AD [2], but among non-demented individuals appears to be primarily associated with decline in memory rather than other cognitive domains or global cognition [33]–[35]. With longer follow-up periods than ours (at least 3 years on average), these studies may have allowed more time for significant levels of memory decline to develop. However, even within the memory domain, a recent study with a population and follow-up period very similar to ours found *APOE* ε4 was associated with decline in visual memory, but not with decline in visual episodic memory, verbal memory, working memory or a visual/working memory composite [36]. Our finding of no association between *APOE* ε4 and decline on a composite memory measure is consistent with this. While *APOE* ε4 reportedly promotes a transition from MCI to AD, its association with MCI itself may be low [4]. This might explain why we found no association between *APOE* ε4 and incident MCI/dementia, as over 80% of our incident cases were MCI. Other measures may have shown no association with cognitive decline in our study because their most salient effects are generated during earlier periods of the lifespan. This includes cholesterol [37] and BMI [38], for both of which late life cognitive impairment has been associated with high levels specifically in mid life.

In some cases the direction of our findings was unexpected, including more educated participants exhibiting greater decline in attention/processing speed. While this seems inconsistent with the concept of cognitive reserve [39], it is similar to previous reports of more educated individuals experiencing greater decline in verbal memory [5] and processing speed [40]. More extensive education may serve to delay the onset of cognitive decline, with this beginning at an earlier age in less educated individuals and missed by our study, or may be associated with higher baseline performance that permits greater absolute levels of decline [5]. Diabetes is generally thought to promote cognitive impairment [41], but we found lower rates of incident MCI or dementia in men with diabetes. This is interesting in the context of reports that diabetes slows the rate of cognitive decline in patients with Alzheimer’s disease [42], [43]. A potential mechanism involving insulin therapy has recently been proposed to account for this effect [44]. Physical activity is generally found to protect against dementia and cognitive decline [45], but we found more active men to have higher rates of incident MCI or dementia. This association may have been biased towards the negative by an effect of study attrition, with men lost to follow-up being less active than men remaining in the study, and other authors reporting participants lost to follow-up as more likely to have developed dementia than those retained [46]. Other reasons for not finding any protective effects of physical activity include a reliance on self-reported activity, with objective measures probably more accurate [47]. Selection for health and survival at follow-up may also explain why men with low health status at baseline exhibited less executive function decline, as a greater proportion of men with low health status were lost to follow-up than remained in the study. Finally, higher rates of low eGFR among individuals lost to follow-up could help explain poor kidney health predicting less memory decline across all participants and a decreased risk of incident MCI or dementia in men.

### Domain Specificity

As reported here, previous studies investigating decline across multiple cognitive domains have found that factors associated with decline on at least one domain are not associated with decline on others [5]–[8]. This is theoretically accounted for by different cognitive processes having different neurological and experiential substrates, and therefore being more or less susceptible to particular factors depending on the mechanisms involved. For example, a previous cross-sectional study found that remitted depression was associated with cognitive deficits primarily involving attention and processing speed [48]. Our finding of greater decline in attention/processing speed for participants with history of depression could be an extension of this (noting though that we also found a more complex history of depression by age interaction for the memory domain). Conversely, we did not identify an expected positive association between physical activity and executive functioning [49], potentially for the methodological reasons already outlined. This highlights the broader problem of considerable variation in how particular domains are reported to be affected by particular factors. For example, at least three other studies [8], [12], [13] have found poorer smell predictive of greater memory decline. Conversely, while we and another study [12] found poorer smell also predictive of executive function decline, a further study did not [13]; and while one study [8] found an association between odor identification and processing speed, we did not. Inconsistency in how tests are assigned to domains likely contributes to this problem (a digit symbol task was assigned to attention/processing speed by us and one other study [8] but to executive functioning by another [13]). This restricts the reliability of domain-specific findings from this and other studies, in addition to limiting the ability to meaningfully compare results across studies. Accordingly, understanding how particular factors affect different types of cognition may be facilitated by future reports including results for individual neuropsychological tests, as an alternative or in addition to those for cognitive domains.

### Population Attributable Risks

Identifying factors that influence cognitive decline is essential for developing interventions. We calculated PARs as indicators of where interventions should be targeted so as to maximise population-wide benefits. The largest PARs were for age. Sex and marital status also had large PARs. Potentially modifiable factors with relatively large PARs included coronary artery disease, arthritis, stroke, and high homocysteine. Redoubling efforts to treat or prevent these conditions would contribute greatly to minimising late-life cognitive decline within the population.

### Limitations

Non-random attrition of less healthy participants and a reliance on self-report measures have already been discussed as limitations of this study. Another limitation is having only a single 2-year follow-up assessment, which invites noise associated with the instability of MCI diagnoses [50] and cognitive performance scores regressing to the mean. Excluding individuals regaining baseline levels of cognitive performance or reverting from MCI to normal cognition after the 2-year follow-up would help address these issues. A 2-year follow-up may also not allow slow acting factors to be identified. These concerns mean that our null and unexpected findings not matching previously reported associations or readily explained by known mechanisms should be treated cautiously. Further limitations of our study include not considering interactions beyond those involving sex and age. Reports of interactions between other factors, like physical activity and *APOE* genotype [51], demonstrate further complexity to the etiology of late-life cognitive decline. Lastly, our results may not be generalizable to all elderly populations, and we expect that different but overlapping factors will emerge in different cohorts. For example, a recent study [7] contrasts with ours in finding that participation in any of mental, physical or social activities protected against decline in one or more cognitive domains. However, the cohort was rural Chinese residents, who differed from our sample on many factors, including much lower levels of education and a far higher proportion of smokers.

### Conclusions and Implications

Highlights of this study are having demonstrated that factors associated with late-life cognitive decline vary with age, sex and outcome measure, and the identification of PARs for which interventions can be applied. The variations in risk and protective factors we found could help explain the inconsistent and poor support for particular factors in the literature. Effects of age and sex may be overlooked when these are adjusted for in statistical procedures rather than investigated as potential interactions or with stratified analyses. These results also suggest that etiologies for cognitive decline can differ between men and women and change over the lifespan. With an aging population, the prevalence and societal burden of cognitive impairment is rapidly increasing. Interventions are urgently required. Our identification of PARs suggests that preventing or treating conditions typical of aging might reduce population-wide levels of cognitive decline and impairment. There may be further benefits in tailoring interventions to particular sex and age groups. Future studies that test relevant interventions are needed to establish the extent to which this is true.

## Supporting Information

Table S1 **PARs for decline in cognitive performance.**
(DOCX)
