# Supplementary material for: Risk Factors for Late-Life Cognitive Decline and Variation with Age and Sex in the Sydney Memory and Ageing Study
Source: PLoS One. 2013 Jun 14;8(6):e65841. doi: 10.1371/journal.pone.0065841 (PMC3683032; doi:10.1371/journal.pone.0065841)
Supplement: Table S1 — PARs for decline in cognitive performance. (DOCX) [file pone.0065841.s001.docx]

**Table S1. PARs for decline in cognitive performance**

| **Cognitive measure** | **Factor** | | **Less severe decline (> 1 SD)** | | |  | **More severe decline (> 1.5 SD)** | | |
| --- | --- | --- | --- | --- | --- | --- | --- | --- | --- |
|  |  |  | **Cases;**  **Controls (No.)** | **OR (95% CI)^a^** | **PAR (%)** |  | **Cases;**  **Controls (No.)** | **OR (95% CI)^a^** | **PAR (%)** |
| **Global cognition** | Age, y | < 81 | 55; 551 | 1.00 (ref) |  |  | 20; 586 | 1.00 (ref) |  |
|  |  | ≥ 81 | 47; 212 | 2.13 (1.37-3.31) | 24.4 |  | 20; 239 | 2.24 (1.11−4.52) | 27.7 |
|  | BSIT score | ≥ 9 | 68; 574 | 1.00 (ref) |  |  | 22; 620 | 1.00 (ref) |  |
|  |  | < 9 | 34; 189 | 1.22 (0.75-1.96) | 5.9 |  | 18; 205 | 1.65 (0.82-3.31) | 17.6 |
|  | Stroke | No | 93; 729 | 1.00 (ref) |  |  | 34; 822 | 1.00 (ref) |  |
|  |  | Yes | 8; 27 | 2.21 (0.94-5.17) | 4.3 |  | 5; 35 | 3.13 (1.07-9.15) | 8.7 |
| **Executive function** | Married | Yes | 70; 373 | 1.00 (ref) |  |  | 37; 406 | 1.00 (ref) |  |
|  |  | No | 46; 278 | 0.88 (0.55-1.40) | −5.5 |  | 19; 305 | 0.63 (0.32-1.24) | −20.2 |
|  | BSIT score | ≥ 9 | 77; 496 | 1.00 (ref) |  |  | 35; 538 | 1.00 (ref) |  |
|  |  | < 9 | 39; 157 | 1.48 (0.94-2.35) | 10.9 |  | 21; 175 | 1.58 (0.84-2.95) | 13.7 |
|  | Homocysteine, μmol/L | ≤ 15 | 83; 543 | 1.00 (ref) |  |  | 39; 587 | 1.00 (ref) |  |
|  |  | >15 | 22; 70 | 1.86 (1.07-3.23) | 9.7 |  | 11; 81 | 1.85 (0.89-3.88) | 10.1 |
| **Attention/PS** | Age, y | < 81 | 76; 517 | 1.00 (ref) |  |  | 29; 564 | 1.00 (ref) |  |
|  |  | ≥ 81 | 59; 186 | 2.24 (1.53-3.28) | 24.2 |  | 22; 223 | 1.99 (1.11-3.55) | 21.4 |
|  | Education, y | ≥ 11 | 79; 389 | 1.00 (ref) |  |  | 25; 443 | 1.00 (ref) |  |
|  |  | < 11 | 56; 314 | 0.84 (0.57-1.24) | −7.7 |  | 26; 344 | 1.29 (0.73-2.31) | 11.6 |
|  | History of depression | No | 110; 603 | 1.00 (ref) |  |  | 41; 672 | 1.00 (ref) |  |
|  |  | Yes | 25; 100 | 1.50 (0.91-2.46) | 6.2 |  | 10; 115 | 1.54 (0.74-3.18) | 6.9 |
| **Memory** | Age, y | < 81 | 54; 544 | 1.00 (ref) |  |  | 11; 587 | 1.00 (ref) |  |
|  |  | ≥ 81 | 54; 195 | 2.57 (1.66-3.97) | 30.5 |  | 20; 229 | 4.31 (1.92-9.64) | 49.5 |
|  | Coronary artery disease | No | 76; 613 | 1.00 (ref) |  |  | 16; 673 | 1.00 (ref) |  |
|  |  | Yes | 32; 126 | 1.61 (0.98-2.66) | 11.2 |  | 15; 143 | 2.64 (1.19-5.88) | 30.1 |
|  | Kidney disease | No | 108; 719 | 1.00 (ref) |  |  | 31; 796 | 1.00 (ref) |  |
|  |  | Yes | 0; 19 | - | - |  | 0; 19 | - | - |
|  | Arthritis | No | 37; 345 | 1.00 (ref) |  |  | 13; 369 | 1.00 (ref) |  |
|  |  | Yes | 70; 386 | 1.80 (1.15-2.81) | 29.1 |  | 17; 439 | 1.04 (0.48-2.25) | 2.1 |
|  | Antidepressants | No | 100; 669 | 1.00 (ref) |  |  | 30; 729 | 1.00 (ref) |  |
|  |  | Yes | 8; 70 | 0.63 (0.27-1.45) | −4.4 |  | 1; 77 | - | - |
|  | Health | ≥ Good | 91; 632 | 1.00 (ref) |  |  | 22; 701 | 1.00 (ref) |  |
|  |  | Low | 17; 105 | 0.95 (0.52-1.73) | −0.8 |  | 9; 113 | 2.17 (0.91-5.18) | 15.6 |
|  | BSIT score | ≥ 9 | 65; 565 | 1.00 (ref) |  |  | 18; 612 | 1.00 (ref) |  |
|  |  | < 9 | 43; 174 | 1.77 (1.12-2.79) | 17.3 |  | 13; 204 | 1.68 (0.75-3.76) | 16.9 |

BSIT = Brief Smell Identification Test; CI = confidence interval; OR = odds ratio; PAR = population attributable risk; PS = processing speed.

^a^ The model for each cognitive outcome contains the factors listed as well as age and sex (if not already present).
